# Expanding our understanding of industry opposition to help implement sugar-sweetened beverage taxation

**DOI:** 10.1017/S1368980021002883

**Published:** 2022-01

**Authors:** Eric Crosbie, Davis Florence

**Affiliations:** 1 School of Community Health Sciences, University of Nevada Reno, 1664 Virginia St., Reno, NV 89557, USA; 2 Ozmen Institute for Global Studies, University of Nevada Reno, Reno, NV, USA

The authors conducted a content analysis to document the strategies, practices and arguments used by the sugar-sweetened beverage (SSB) industry to oppose an SSB taxation proposal in Brazil. This case study represents an important initial step in providing much-needed evidence within a limited literature documenting industry opposition to SSB tax proposals. Given the limited case studies on the issue, it is important to build upon this research by understanding commercial actors as vectors of disease, examining multiple approaches to documenting industry opposition, and most importantly developing counter strategies to mitigate industry opposition. In doing so, this approach contextualises these findings within the emerging field of commercial determinants of health to help provide academics, advocates and policymakers the necessary tools to implement SSB taxation policies.

## Understanding the commercial vector of disease

Before exploring the strategies, practices and arguments of the SSB industry to oppose SSB tax proposals, it is important to understand the SSB industry itself and its impact on public health. In contrast to infectious diseases, such as COVID-19, which arise from an animal vector, most non-communicable diseases (NCD), including diabetes, cancer and CVD, arise from a human-made vector: the transnational corporation. This approach centres around the commercial determinants of health, in which health-harming industries (e.g. tobacco, ultra-processed food and drink, alcohol, pharmaceutical and fossil fuel) are the vectors of disease by promoting products and choices that are detrimental to health^(1)^. Given the primary focus on transnational corporations in the commercial determinants of health literature, one of the strengths of the study is classifying the opposing stakeholders into two prominent industry trade associations. One represents the transnational food and beverage corporations and the other represents the main sugar, ethanol and bioelectricity producers. Examining these various stakeholders that make up the SSB industry, and food and beverage industry more broadly, provides a deeper understanding of the commercial vector of disease. For example, in Colombia, sugar production comprises of approximately two-thirds of the total water footprint or soda and depending on the sweetener it takes an estimated 442–618 l of fresh water to produce 1 l of soda^(2)^. These aspects have important implications for regulating sugar consumption.

## Multiple approaches to understanding SSB industry opposition

Studying any vector of disease requires multiple approaches to examine the causation and determinants of diseases. The authors employ the corporate political activity framework which describes a set of strategies used by the industry to influence public health practices. These practices revolve around a set of strategies involving information/messaging, financial incentives, constituency building, policy substitution, legal approaches, and constituency fragmentation/destabilisation. The authors found evidence of ‘information and messaging” (industry dissemination of information) and ‘policy substitution’ (voluntary or self-regulation when threatened by government regulations) when analysing public hearings in Brazil. They also discovered an additional practice ‘stress the importance of the environment’ from the sugar cane industries. This has important implications for countries where sugar cane is the main source of sugars for the industry and increasing sugar cane crops raises environmental concerns due to its intensive use of water and deforestation^(3)^.

Another commonly applied approach to understand industry opposition to public health policy proposals such as SSB taxation is applying the policy dystopia model, which analyses industry discursive (argument-based) strategies and instrumental (action-based) strategies^(4)^. This approach provides another taxonomy of classifying common industry strategies similar to the corporate political activity framework. Both approaches illustrate how industries use similar strategies to avoid regulation across different countries and settings. For example, common industry arguments found in Brazil include: SSB taxes are regressive, disproportionally impact low-income communities and drive job losses mirroring similar arguments employed in countries such as Ireland, South Africa and Mexico^(5)^.

Another approach, not as commonly applied, is examining industry structural strategies, which alter the rules, procedures and practices that make regulatory environments more conducive for industry opposition to block public health proposals such as SSB tax increases. In the USA, the SSB industry has used state preemption, the use of state law to limit and restrict local authority, to prevent localities from enacting local SSB taxes. This practice dramatically diminishes discussion and debate and social norm change, causes a chilling effect on other localities seeking to enact similar taxes^(6,7)^, and once these policies are in effect they are extremely difficult to repeal^(8)^. Globally, the SSB industry has also lobbied trade negotiators to structurally alter trade rules that have effectively constrained policymakers from implementing public health policies^(9,10)^. This includes the usage of the World Trade Organization and Codex Alimentarius, which establishes international standards and guidelines relating to food production and food safety. While to date this has not directly impacted SSB taxes, the SSB industry has used international treaties to globally preempt policies aimed at reducing sugar consumption such as front-of-pack nutrition labelling^(11–13)^.

## Cross-industry ties and comparisons

The authors use a standard case study approach focusing on analysing public hearings on a single policy issue of SSB taxes in Brazil. Future research in this area should compare industry strategies across sectors, issue areas and venues. It is no secret that transnational corporations in many industries including tobacco, alcohol and food have close ties and use similar strategies in terms of political lobbying, corporate marketing, corporate social responsibility initiatives and supply chain management^(1)^. Yet, we still lack empirical research in comparing these industry ties and practices across sectors. Social discourse analysis, which combines political framing and social network analyses using publicly available media sources, is a recent approach that offers an effective strategy for mapping these practices across sectors. Social discourse analysis was initially used to compare two fiscal policy debates: Scotland’s minimum-pricing policy for alcohol and the United Kingdom’s sugar tax on soft drinks^(14)^. However, social discourse analysis, along with the CPA framework and the policy dystopia model, can be further extended^(15)^ by examining: (1) a broader range of industry vectors (pharmaceutical, fossil fuel and firearms); (2) various policy debates (marketing and advertising, and labelling and health warnings); (3) predominant narratives in policy debates over time (cross-industry deflection of industry responsibility onto individual consumers)^(16)^; (4) policies in low- and middle-income countries where NCD are rapidly growing, representing 80 % of deaths^(17)^; and (5) the diffusion of best practices as industries aggressively attempt to limit diffusion^(18)^. These approaches can not only help reveal common strategies and clusters of stakeholders that share cross-industry ties but can increase knowledge sharing, collaboration and coalition building across currently isolated public health stakeholders^(15)^.

## Exploring the other side: countering industry strategies and debunking their myths

To fully understand the adoption or rejection of SSB tax proposals, it requires not only analysing industry barriers but also how to counter them to ensure taxation implementation. This involves analysing the role of supporting stakeholders such as local and international public health organisations which are supported by environmental groups, philanthropic donors, inter-governmental organisations, and health economists and lawyers. These separate groups can help form transnational networks and coalitions capable of pooling financial resources and technical expertise to combat powerful industry actors^(19)^. In particular to the SSB tax debates, it is important for supporting stakeholders to collaborate and expose industry tactics and debunk industry myths. First, the SSB industry wants to be ‘part of the solution’ working with health groups and government through voluntary self-regulations and public–private partnerships^(20)^. Similar to tobacco and alcohol, these suggested solutions are non-transparent and have a history of being ineffective at reducing NCD. Second, the SSB industry attempts to frame the problem of the NCD epidemic based on individual/personal or parental responsibility. It should be noted that the industry recruits physical activity scholars to build this narrative with a strong emphasis on physical activity and unhealthy diets shifting the blame away from the consumption of their unhealthy products^(21)^. Yet, unhealthy food and drinks drive unhealthy diets^(22)^, and the SSB industry has also sponsored research to spread misinformation and create doubt about the harmfulness of its products^(21)^. Third, the SSB industry claims proponents view SSB taxes as a silver bullet (simple but sweeping solution) to obesity and diet-related health problems. However, proponents in Brazil and in other countries typically advocate for a set of recommendations, including advertising restrictions and front-of-pack nutrition labelling that should be implemented in combination to reduce NCD. Fourth, the industry attempts to cast doubt on the effectiveness of taxing SSB arguing these policies are regressive, disproportionally impact low-income communities, drive job losses and increase expenses to consumers with no improvement in public health^(23)^. These arguments, however, have largely been refuted by research. SSB taxes often benefit low-income communities when revenues are reinvested to build health equity in these communities; they do not create job losses (in some cases jobs in the food sector increase following SSB taxes)^(24)^, and they improve public health by reducing sales of these unhealthy beverages^(25)^. In summary, expanding our understanding of commercial determinants of health, identifying industry strategies and adopting best practices to counter industry opposition will help implement these policies and ultimately help curb the NCD epidemic and save lives.
